# Ginkgolide B Maintains Calcium Homeostasis in Hypoxic Hippocampal Neurons by Inhibiting Calcium Influx and Intracellular Calcium Release

**DOI:** 10.3389/fncel.2020.627846

**Published:** 2021-02-10

**Authors:** Li Wang, Quan Lei, Shuai Zhao, WenJuan Xu, Wei Dong, JiHua Ran, QingHai Shi, JianFeng Fu

**Affiliations:** ^1^Clinical Laboratory Diagnostic Center, General Hospital of Xinjiang Military Command, Urumqi, China; ^2^The Department of Medical Administration, General Hospital of Xinjiang Military Command, Urumqi, China; ^3^The First Division Health Team, Anti-aircraft Artillery of Liaoning Reserve, Shenyang, China

**Keywords:** ginkgolide B, hypoxia, cytoplasmic calcium, neuron, homeostasis

## Abstract

Ginkgolide B (GB), a terpene lactone and active ingredient of *Ginkgo biloba*, shows protective effects in neuronal cells subjected to hypoxia. We investigated whether GB might protect neurons from hypoxic injury through regulation of neuronal Ca^2+^ homeostasis. Primary hippocampal neurons subjected to chemical hypoxia (0.7 mM CoCl_2_) *in vitro* exhibited an increase in cytoplasmic Ca^2+^ (measured from the fluorescence of fluo-4), but this effect was significantly diminished by pre-treatment with 0.4 mM GB. Electrophysiological recordings from the brain slices of rats exposed to hypoxia *in vivo* revealed increases in spontaneous discharge frequency, action potential frequency and calcium current magnitude, and all these effects of hypoxia were suppressed by pre-treatment with 12 mg/kg GB. Western blot analysis demonstrated that hypoxia was associated with enhanced mRNA and protein expressions of Ca_v_1.2 (a voltage-gated Ca^2+^ channel), STIM1 (a regulator of store-operated Ca^2+^ entry) and RyR2 (isoforms of Ryanodine Receptor which mediates sarcoplasmic reticulum Ca^2+^ release), and these actions of hypoxia were suppressed by GB. Taken together, our *in vitro* and *in vivo* data suggest that GB might protect neurons from hypoxia, in part, by regulating Ca^2+^ influx and intracellular Ca^2+^ release to maintain Ca^2+^ homeostasis.

## Introduction

A hypoxic environment can severely damage organs, especially the brain. Numerous investigations have provided evidence that a hypoxic environment can induce oxidative stress (Ramanathan et al., 2005; Niki, 2010; Yan et al., 2019), apoptosis (Li et al., 2007; Leszczynska et al., 2015), inflammation (Yang et al., 2013, 2015; Koh et al., 2015), autophagy (Xu and Zhang, 2011; Yang et al., 2015) and Ca^2+^ overload (Zhao et al., 1999) among other deleterious effects. Therefore, efforts are underway to develop new strategies to attenuate or prevent the damage caused by hypoxia, and there has been recent interest in the use of alternative medicines to antagonize hypoxia-induced tissue damage, such as aminophylline (Yang et al., 2007) diazepam, dexamethasone (Gong et al., 2015), choline (Zhang et al., 2017) and Rhododendron simsii Planch flower (Guo et al., 2020).

*Ginkgo biloba* is a tree native to China that is used in traditional medicine. Previous research has indicated that *Ginkgo biloba* leaf extracts or active components may be beneficial for the prevention of hypoxic injury, likely via anti-inflammatory, anti-apoptotic and antioxidant activity (Karcher et al., 1984; Jowers et al., 2004; Han and Li, 2013). Ginkgolide B (GB) is a terpene lactone and important active ingredient of *Ginkgo biloba* (DeFeudis and Drieu, 2000). Notably, numerous studies have demonstrated that GB exerts protective actions in animal models of cerebral ischemia and reperfusion (Lv et al., 2011; Shah et al., 2011; Gu et al., 2012; Huang et al., 2012; Shu et al., 2016; Wei et al., 2017), indicating that GB has potential as a therapy to ameliorate the effects of ischemic stroke. However, the mechanisms underlying the beneficial effects of GB in the setting of cerebral hypoxia remain to be fully characterized.

Recent studies have focused on the possible role of GB in the regulation of intracellular Ca^2+^, which functions as a second messenger in signal transduction pathways in mammalian cells (Carafoli and Krebs, 2016). In the cardiovascular system, GB can non-competitively suppress the vasopressor effect of serotonin *in vitro* and selectively inhibit serotonin-mediated Ca^2+^ mobilization in vascular smooth muscle cells (Wang et al., 2002). Furthermore, it has been suggested that GB might exert a cardioprotective effect via the regulation of Ca^2+^ signaling pathways, Akt and reactive oxygen species (Gao et al., 2016). Other experiments found that GB could partially protect PC12 cells from 6-hydroxydopamine-induced apoptosis through an upregulation in the expression of calbindin D28K mRNA as well as a decrease in intracellular Ca^2+^ concentration (Meng et al., 2007). Additionally, *Gingko biloba* extract has been reported to suppress agonist-induced Ca^2+^ signaling in endothelial cells (Campos-Toimil et al., 2000). However, the effects of GB on intracellular Ca^2+^ regulation in cerebral neurons are presently unknown.

We hypothesized that GB might protect against hypoxia-induced neuronal injury in part through effects on intracellular Ca^2+^ signaling. Therefore, the aim of this study was to investigate whether GB suppressed the effects of hypoxia on rat hippocampal neurons by regulating Ca^2+^ influx and intracellular Ca^2+^ release to maintain Ca^2+^ homeostasis.

## Materials and Methods

### Establishment of an *in vitro* Model of Hypoxia in Cultured Hippocampal Cells

Sprague-Dawley male rats aged 1–3 days were provided by the Animal Center of the Fourth Military Medical University (Xi'an, China). All procedures were conducted in strict accordance with the National Institutes of Health Guide for the Care and Use of Laboratory Animals (National Institutes of Health Publications, No. 80–23, revised 1978) and approved by the Ethics Committee for Animal Experiments of the Fourth Military Medical University.

Briefly, rats were euthanized, and the hippocampi were resected under a stereoscopic microscope and placed in Mg^2+^- and Ca^2+^-free ice-cold Hank's Balanced Salt Solution (HBSS; cat. #H4385, Seebio Biotech, Shanghai, China). Hippocampi were incubated in 0.25% trypsin (cat. #15050065, Gibco, Thermo Fisher Scientific, Rockford, IL, USA) for 10 min at37°C, and digestion was then inactivated with fetal bovine serum (FBS: cat. #10099, Gibco, Thermo Fisher Scientific, Rockford, IL, USA). The tissue solution was centrifuged at 1,000 rpm for 5 min, and cells were cultured in Mg^2+^- and Ca^2+^-free HBSS containing 1% streptomycin/penicillin (cat. #15140163, Gibco, Thermo Fisher Scientific, Rockford, IL, USA) and 10% FBS in a humidified environment with 5% CO_2_ at 37°C for 6 h. Subsequently, the cells were transferred into neurobasal medium (cat. #10888-022, Gibco, Thermo Fisher Scientific, Rockford, IL, USA) containing 2% B27 (cat. #A1486701, Gibco, Thermo Fisher Scientific, Rockford, IL, USA), 10% FBS, 1% streptomycin/penicillin and 1% L-glutamine (cat. #21051024, Gibco, Thermo Fisher Scientific, Rockford, IL, USA). One-half of the culture medium was changed every 3 to 4 days without glutamate. Cells were used between 11 and 13 days in culture until 70–80% confluence was reached.

Chemical hypoxia was treated by CoCl_2_ (Muñoz-Sánchez and Chánez-Cárdenas, 2019). The cultured hippocampal neurons were divided into four experimental groups: (1) control group (cultured in normal medium); (2) GB group (cultured in normal medium and incubated with 0.4 mM GB (cat. #15291-77-7, LOT: C31J6G2014, Purity >98%, Shanghai Yuanye CO, Ltd, China) for 24 h); (3) hypoxia group (treated with 0.7 mM CoCl_2_ (cat. #C8661, Sigma-Aldrich, USA) in FBS-free medium for 12 h to induce chemical hypoxia); and (4) hypoxia+GB group (incubated in normal medium containing 0.4 mM GB for 24 h followed by FBS-free medium containing 0.7 mM CoCl_2_ and 0.4 mM GB for 12 h).

### Establishment of an *in vivo* Model of Hypoxia

Adult (4-week-old) male Sprague-Dawley rats were maintained in a temperature- and humidity-controlled environment in a 12-h/12-h dark/light cycle with free access to water and food. All procedures were conducted in accordance with the National Institutes of Health Guide for the Care and Use of Laboratory Animals (National Institutes of Health Publications, No. 80–23, revised 1978) and approved by the Ethics Committee for Animal Experiments of the Fourth Military Medical University. Rats were randomly assigned to four experimental groups: control group (*n* = 18), GB group (*n* = 18), hypoxia group (*n* = 18) and hypoxia+GB group (*n* = 18).

Rats in the control group were kept in a standard environment for 7 days. Rats in the GB group received intragastric administration of GB (12 mg/kg) every day for 7 days while being maintained under the same conditions as those in the control group. Rats in the hypoxia group were exposed to simulated hypobaric hypoxia at 6,000 m for 7 days in an animal decompression chamber (FLYDWC50-IA, Aviation Industry Corporation of China, Beijing, China) with the temperature and humidity maintained at 20 ± 2°C and 30 ± 5%, respectively. The rate of ascent to simulated high altitude was 40 m/sec, and pressure was maintained at 354 ± 2 mmHg. Fresh air was allowed to flow into the chamber at a rate of 5.5 L/min during the exposure to hypoxia. Rats in the hypoxia+GB group received intragastric administration of GB (12 mg/kg) for 3 days before and the first 2 days during exposure to hypoxia in the decompression chamber (7 days at a simulated 6,000 m).

### Confocal Fluorescence Microscopy

Cultured cells were washed with HBSS three times and incubated with 5 μM fluo-4 AM (cat. #F312, Dojindo, Kumamoto, Japan) in Ca^2+^-free medium (10 mM HEPES, 2 mM MgCl_2_, 2 mM KCl, 35 mM NaCl and 4 g/L glucose) for 45 min at 37°C in the dark. The cells were then incubated in fresh Ca^2+^-free medium (without fluo-4 AM) for 20 min in the dark (Xia et al., 2009). A confocal laser-scanning microscope (FV1000, Olympus, Tokyo, Japan) was used for observation and imaging of intracellular fluo-4 fluorescence (excitation at 488 nm provided by an argon laser), and FluoView software (Olympus) was used to capture digital images. Relative intracellular free Ca^2+^ concentration ([Ca^2+^]_i_) was calculated as F / F_0_, where F_0_ was the baseline fluorescence, and F was the measured fluorescence when the experiment approached its end. Image capture was performed every 10 s for a total of 20 min, and data were collected from eight cells for each group.

### Brain Slice Preparation

Rats were killed by decapitation, and the ventral hippocampi were resected, cut flat and placed on agar using adhesive. The brain was sectioned (400 μm) at a temperature of 0°C using a vertical vibratome (VT1200s, Leica, Wetzlar, Germany) in sucrose cutting solution containing (in mM): CaCl_2_ 0.5, MgSO_4_·7H_2_O 6, sucrose 252, NaHCO_3_ 26, NaH_2_PO_4_ 1.2, KCl 2.5, and glucose 10, aerated with 95% O_2_/5% CO_2_ (pH 7.4). Prior to electrophysiological recording, brain slices were equilibrated for 2 h at room temperature in a submerged recovery chamber with oxygenated artificial cerebrospinal fluid containing (in mM): CaCl_2_ 2, NaHCO_3_ 25, NaCl 124, MgSO_4_·7H_2_O 6, NaH_2_PO_4_ 1, KCl 2.5, and glucose 37. All drugs used in these experiments were purchased from Sigma-Aldrich (St. Louis, MO, USA) or Invitrogen (Carlsbad, CA, USA).

### Electrophysiology

The conventional whole-cell voltage-clamp technique was adopted for the electrophysiological studies, which were performed using a MultiClamp 700B patch-clamp amplifier (Axon Instruments, Union City, CA, USA) and a patch electrode created from borosilicate glass using a micropipette puller (model P-97, Sutter Instruments, Novato, CA, USA). pCLAMP software (Version 10.0, Axon Instruments) was used to acquire and analyze the data.

Recordings of spontaneous discharges and action potentials were made using patch pipette solution containing (in mM): Tris-GTP 0.2, Mg-ATP 4, HEPES 10, EGTA 0.4, KCl 15, NaCl 5, and K-gluconate 130, pH 7.25–7.35. The resistance of the patch electrode was typically 3.5–4.5 MΩ. Neurons (*n* = 9) were clamped at−60 mV and allowed to equilibrate for at least 3 min prior to data collection. Then, currents were recorded for 5 min. Action potentials were stimulated by the injection of 400-ms depolarizing current steps from 0 to 80 pA in 10-pA increments.

Ca^2+^ current (*I*_*Ca*_) was elicited by 200-ms depolarizing steps to −50 mV. For analysis of the current-voltage (I-V) relationship, 200-ms depolarizing voltage steps were applied to a range of potentials from −50 mV to +20 mV (10 mV increments). Tetrodotoxin (TTX, 30 μM) and tetraethyl ammonium (TEA, 40 μM) were added to the extracellular solution to block Na^+^ and K^+^ currents, respectively. The recorded currents could be completely blocked by CdCl_2_ (20 μM), which is known to block Ca^2+^ currents. The solution used to fill the patch pipette contained (in mM): CsCl 150, HEPES 5, EGTA 10, MgCl_2_ 1, ATP-Na_2_ 5, creatine phosphate Na_2_ 5 (pH 7.2). The extracellular solution contained (in mM): Tris 75, BaCl_2_ 50, HEPES 10, glucose 10 (pH 7.4). All recordings were made at 35–37 °C.

### Western Blot Assay

The rats were deeply anesthetized and perfused with 0.9% saline followed by 4% paraformaldehyde. The hippocampi were dissected, and the tissue was lysed with 300 μL lysis buffer containing (in mM): EDTA 1, 0.5% NP-40, 1% Triton X-100, NaCl 150, and Tris 10 (pH 7.4). Protein was quantified using a bicinchoninic acid assay (Thermo Fisher Scientific). Sodium dodecyl sulfate-polyacrylamide gel electrophoresis was used to resolve 30 μg of the cell lysates, which were then transferred onto polyvinylidene difluoride membranes (Immobilon-P, Millipore, Thermo Fisher Scientific). The membranes were blocked for 1 h in non-fat milk and then incubated overnight at 4°C with the following primary antibodies: rabbit anti-Ca_v_1.2 (1:200, cat. #ACC-005, Alomone, Jerusalem, Israel), rabbit anti-stromal interaction molecule-1 (STIM1; 1:500, cat. #ACC-063, Alomone), rabbit anti-ryanodine receptor type-1 (RyR2; 1:200, cat. #ARR-001, Alomone) and rabbit anti-β-actin (1:2,000, cat. #4970, Cell Signaling Technology, Danvers, MA, USA). After washing, membranes were incubated with horseradish peroxidase-conjugated secondary antibodies (anti-rabbit, 1:5,000, cat. #9811, Amersham Pharmacia Biotech, Amersham, UK). An enhanced chemiluminescence detection method (Amersham Pharmacia Biotech) was used to detect each protein, and membranes were visualized by exposure to film. The films were scanned, and ImageJ software (National Institutes of Health) was used to quantify and analyze the images. Target protein levels were normalized to β-actin levels, and the data are expressed as fold-change relative to the control group.

### Quantitative PCR (qPCR)

Total RNA was extracted from the hippocampal homogenate with a total RNA extraction kit (cat. #9767, Takara Bio, Shiga, Japan), and the PrimeScript® 1st Strand cDNA Synthesis Kit (cat. #D6110A, Takara Bio) was used for cDNA synthesis. The expressions of genes related to Ca^2+^ influx and intracellular Ca^2+^ release were quantified using the following specific primers:

5′-GATGCAAGACGCTATGGGCTATGA-3′,

5′-GCATGCTCATGTTTCGGGGTTTGTC-3′ for Ca_v_1.2;

5′-GGCCAGAGTCTCAGCCATAG-3′,

5′-TAGTCGCACCTCCTGGATAC-3′ for STIM1;

5′-GGCCATCCTTGTCCAGCATTAC-3′,

5′-CTGCTCCGTAATGTAAAGCCCATC-3′ for RyR2;

The following conditions were used for thermal cycling: 30 cycles for 15 s at 94°C, 30 s at 55°C, and 30 s at 72°C; 1 cycle for 10 min at 72°C. The PCR reactions were performed using an SYBR green-based system (cat. #RR82LR, Takara), and the gene fold-changes were calculated using the 2^−ΔΔCt^ method.

### Cell Viability Assay

Cell viability was evaluated by MTT assays as previously described (Denizot and Lang, 1986). Dissociated cells were plated at a density of 4× 10^3^ cells/well onto 96 wells previously. Afterwards, supernatants were removed and cells were solubilized with DMSO (cat. #D12345, Gibco, Thermo Fisher Scientific, Rockford, IL, USA) to detect the crystals formed in the viable cells. The cell viability is expressed as a percentage of the OD (*A*570 nm) of cells. The groups and pre-treatments were same as *in vitro* model of hypoxia.

### Statistical Analysis

The analyses were performed using SPSS 15.0 (SPSS Inc., Chicago, IL, USA). Data are expressed as the mean ± standard error of the mean (SEM). Comparisons between two independent groups were made using Independent Sample *t*-test. Comparisons between multiple groups were made using one-way or two-way analysis of variance (ANOVA) followed by the Bonferroni *post-hoc* test. *P* < 0.05 was considered significant.

## Results

### GB Enhances the Cellular Metabolic Activity of Neurons Exposed to Chemical Hypoxia *in vitro*

**Figure 1:**
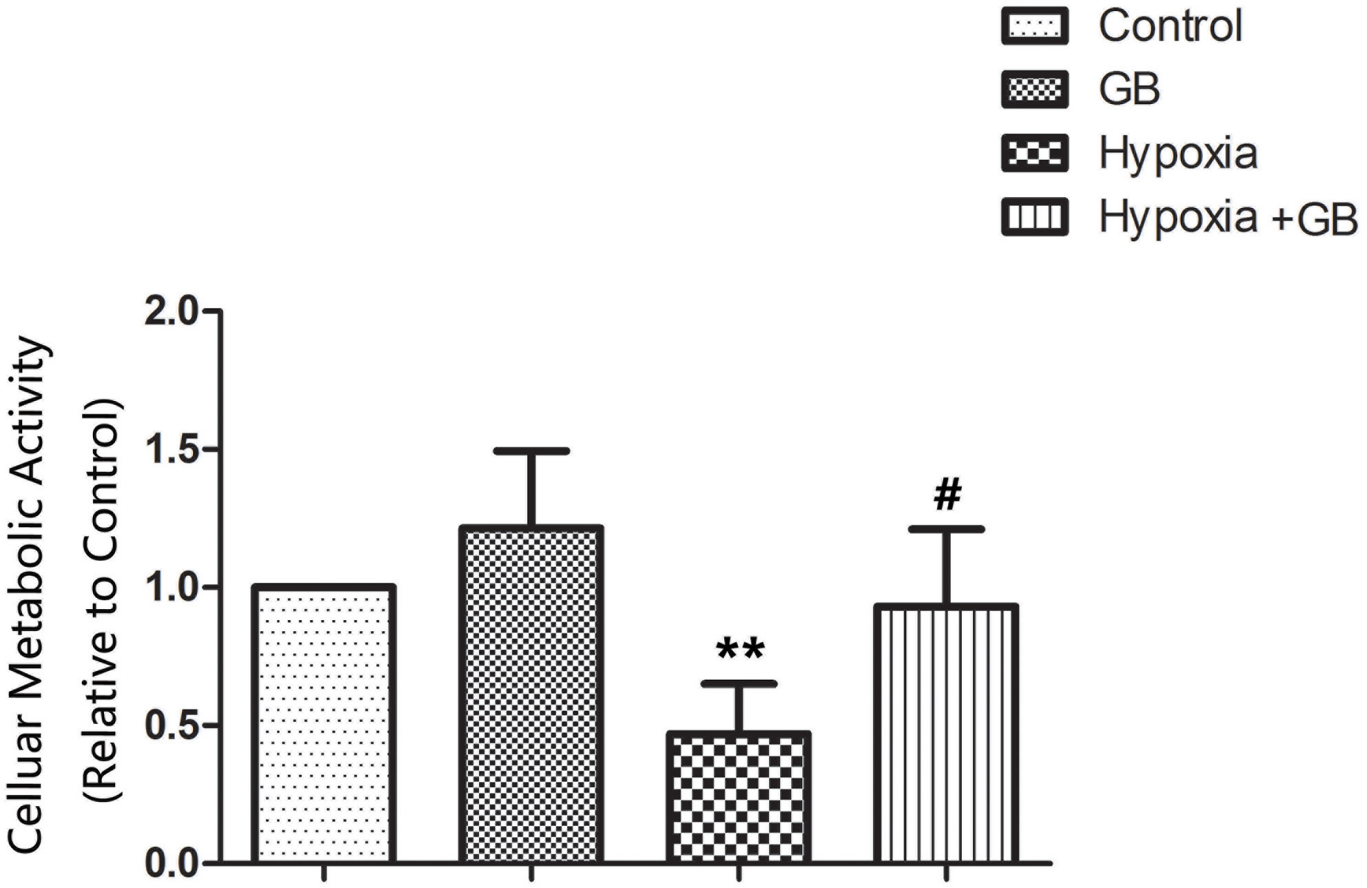
GB increased the cellular metabolic activity of cultured neurons under hypoxic conditions. Chemical hypoxia was induced in cultured primary hippocampal neurons by treatment with 0.7 mM CoCl_2_ for 12 h. 0.4 mM GB was administered for 24 h before and during exposure to CoCl_2_. Data are shown as the mean ± SEM. **P < 0.01 vs. control group; ^#^*P* < 0.05 vs. hypoxia group (Student's *t*-test).

Chemical hypoxia (treatment with CoCl_2_) significantly reduced the viability of primary hippocampal neurons cultured *in vitro* (*P* < 0.01 vs. control group; Figure 1). However, GB significantly increased the cellular metabolic activity of neurons exposed to chemical hypoxia (*P* < 0.05 vs. hypoxia group; Figure 1), indicating that it protected neurons against hypoxic injury. The administration of GB alone (in the absence of chemical hypoxia) did not affect cell cellular metabolic activity (Figure 1).

### GB Attenuates the Increase in Neuronal [Ca^2+^]_i_ During Hypoxia *in vitro*

**Figure 2:**
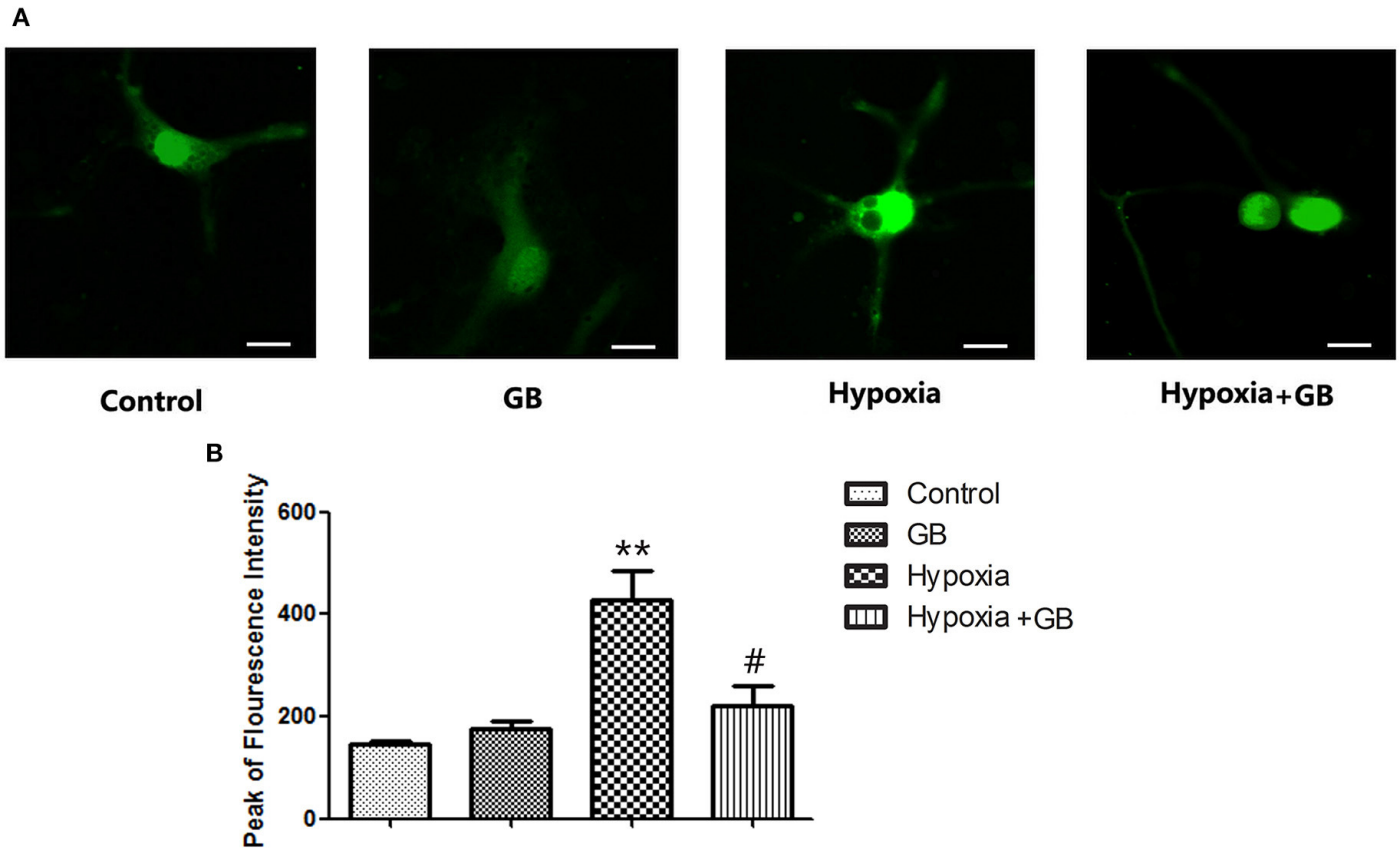
GB decreased the [Ca^2+^]_i_ of cultured neurons under hypoxic conditions. **(A)** Representative images showing fura-4 fluorescence (green; used as a measure of [Ca^2+^]_i_) in cultured primary hippocampal neurons from each of the four experimental groups. Scale bar: 10 μm. **(B)** Quantification of the fluorescence intensity in the control, GB, hypoxia and hypoxia+GB groups. Data are expressed as the mean ± SEM (*n* = 8). **P < 0.01 vs. control group; ^#^*P* < 0.05 vs. hypoxia group (Student's *t*-test).

Representative images showing fluo-4 fluorescence (indicating [Ca^2+^]_i_) in cultured primary hippocampal neurons from the four experimental groups are presented in Figure 2A. Fluo-4 fluorescence intensity was markedly higher in the hypoxia group than in the control group (*P* < 0.01, Figure 2B) but significantly lower in the hypoxia+GB group than in the hypoxia group (*P* < 0.05, Figure 2B). Meanwhile the administration of GB alone did not affect the Fluo-4 fluorescence intensity. These results suggest that hypoxia increases [Ca^2+^]_i_ in neurons and that treatment with GB inhibits or reverses this effect through presently unclear mechanisms. Because these experiments were conducted in Ca^2+^-free medium, the observed changes in [Ca^2+^]_i_ likely reflect alterations in Ca^2+^ release from mitochondrial and/or endoplasmic reticulum (ER) Ca^2+^ stores.

### GB Inhibits the Excitability and Ca^2+^ Currents of Hypoxic Neurons *in vivo*

**Figure 3:**
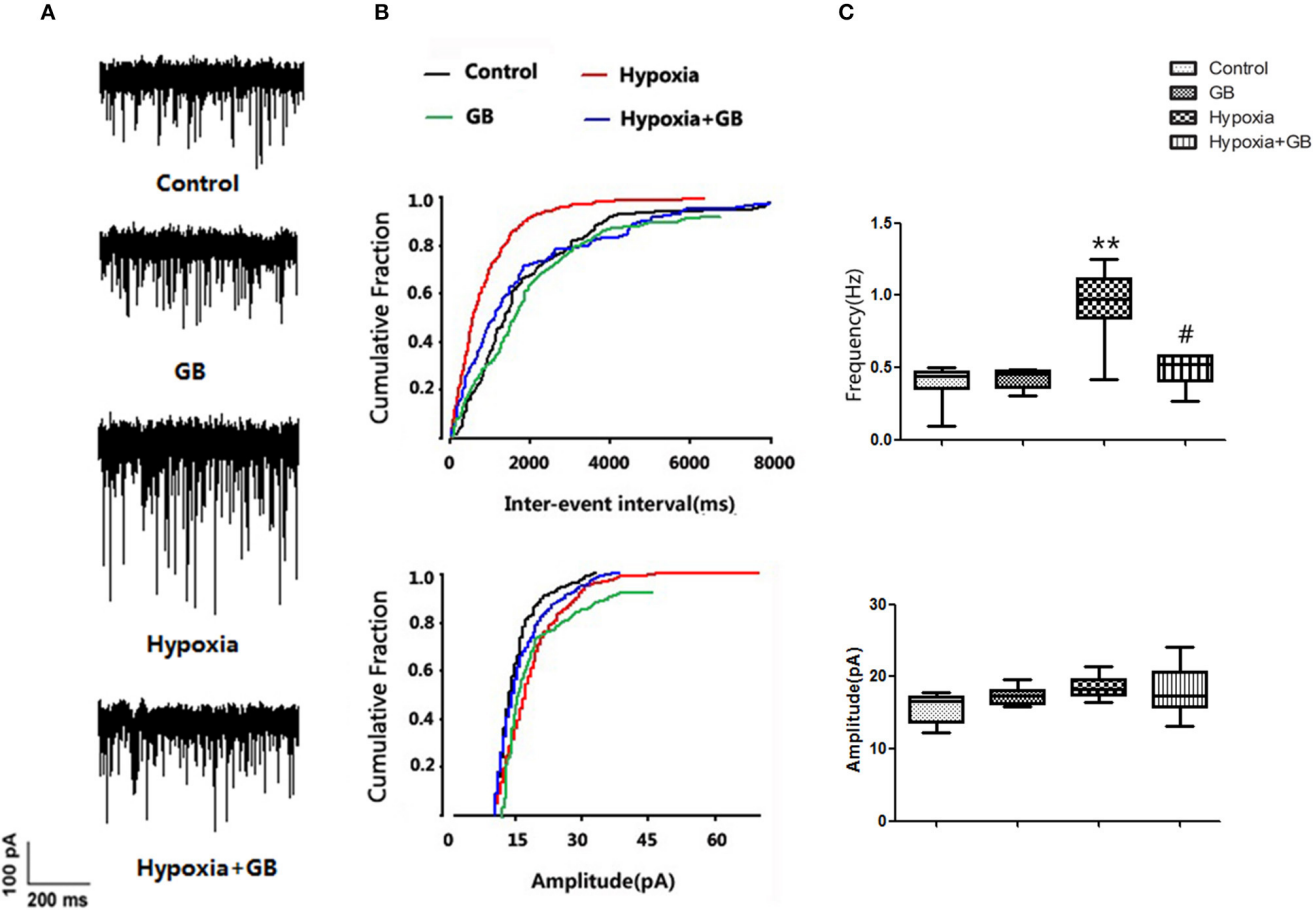
GB inhibited the post-synaptic excitability of hippocampal neurons exposed to hypoxia *in vivo*. **(A)** Representative traces showing spontaneous discharges reflecting spontaneous excitatory post-synaptic potentials (sEPSCs) recorded from hippocampal slices obtained from the control, GB, hypoxia and hypoxia+GB groups. **(B,C)** Summary results for the effects of GB on sEPSCs. Data in **(C)** are expressed as the mean ± SEM (*n* = 9). **P < 0.01 vs. control group; ^#^*P* < 0.05 vs. hypoxia group (Student's *t*-test).

We next examined whether GB influenced the effects of hypoxia on neuronal excitability *in vivo*. As shown in Figure 3, hypoxia was associated with a significant increase in spontaneous discharge frequency (Control group: 0.38 ± 0.11; Hypoxia group: 0.87 ± 0.23; *P* < 0.05), indicating that neuronal excitability was elevated. Moreover, the hypoxia-induced enhancement of spontaneous discharge frequency was significantly attenuated by GB (Hypoxia group, 0.87 ± 0.23; Hypoxia+GB group, 0.53 ± 0.13; *P* < 0.05), suggesting that GB decreased neuronal excitability during hypoxia. There were no significant differences between groups in the mean amplitude of the spontaneous discharge (Figure 3). We also found that treatment only with GB did not affect the spontaneous discharge frequency and the amplitude of the spontaneous discharge (Figure 3).

**Figure 4:**
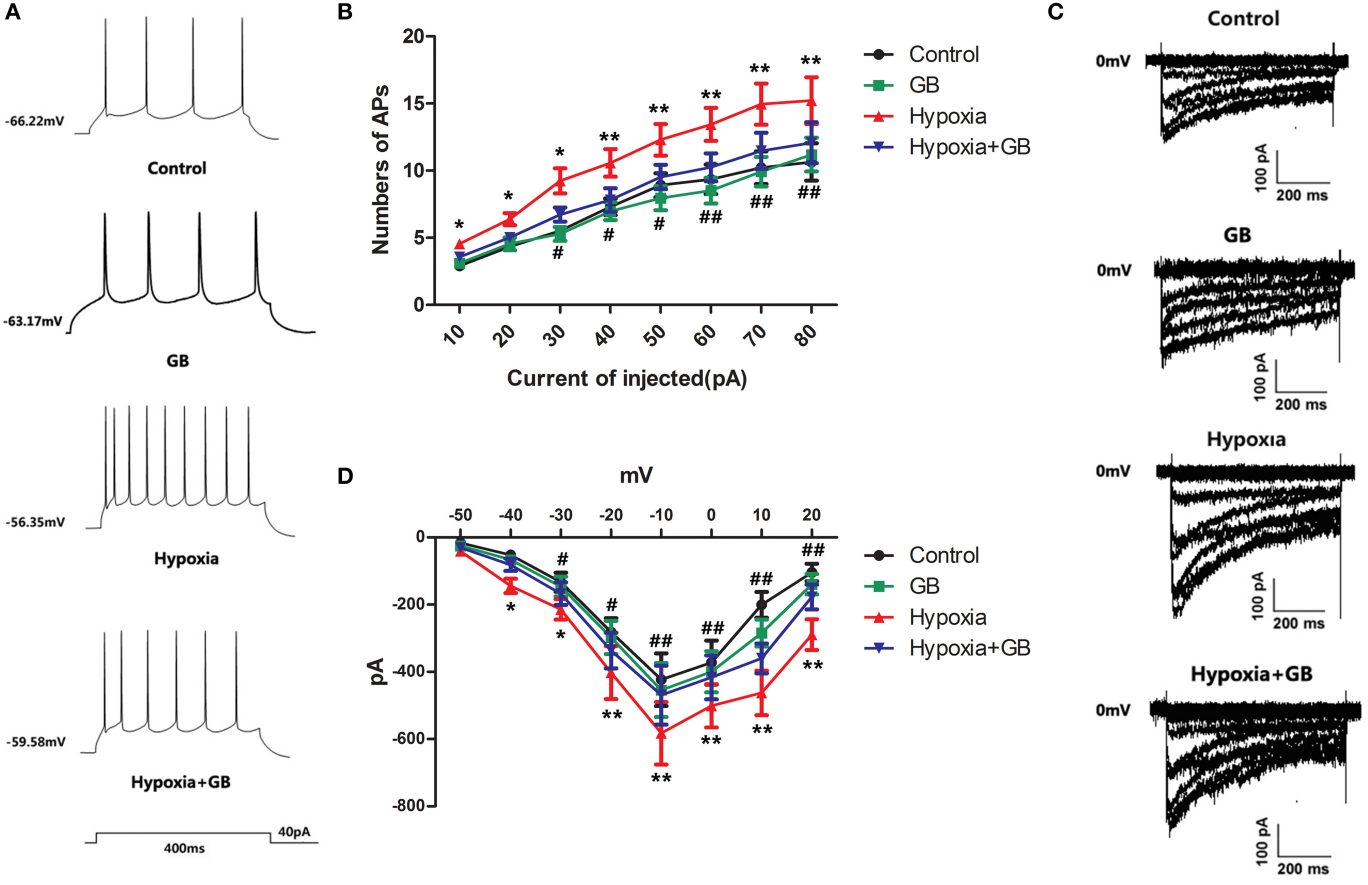
GB reduced calcium currents in hippocampal neurons exposed to hypoxia *in vivo*. **(A)** Representative traces showing action potentials recorded from hippocampal neurons during the injection of a 40-pA depolarizing current for 400 ms. **(B)** Summary data for the number of action potentials (APs) recorded for each injected current amplitude for neurons in the control, GB, hypoxia and hypoxia+GB groups. **(C)** Representative traces showing calcium currents recorded from neurons in the control, GB, hypoxia and hypoxia+GB groups. **(D)** Current-voltage (I-V) curves obtained for neurons in the control, GB, hypoxia and hypoxia+GB groups. Data in **(C)** and **(D)** are expressed as the mean ± SEM (*n* = 9). *P < 0.05, **P < 0.01 for hypoxia group vs. control group; ^#^*P* < 0.05, ^##^*P* < 0.01 for hypoxia+GB group vs. hypoxia group (one-way ANOVA and Bonferroni *post-hoc* test).

We next examined the effects of GB on the action potentials of hypoxic neurons. The current-clamp mode was used to record neuronal action potentials elicited by the injection of depolarizing current of varying amplitude (0, 10, 20, 30, 40, 50, 60, 70, and 80 pA) for 400 ms (Figure 4A). Compared to neurons in the control group, there was an obvious increase in the number of action potentials in neurons in the hypoxia group (*P* < 0.05 or 0.01 for all current amplitudes; Figure 4B). Furthermore, pre-treatment with GB was associated with significant reductions in the number of action potentials under hypoxic conditions (*P* < 0.05 or 0.01 for current amplitudes of 30–80 pA; Figure 4B). Taken together, these results suggest that GB suppresses or inhibits neuronal excitability under hypoxic conditions.

Finally, we used the voltage-clamp technique to record neuronal Ca^2+^ currents (*I*_*Ca*_). Calcium current (*I*_*Ca*_) was evoked by step depolarization from −80 to 0 mV, and calcium currents started to activate at −40 mV. As shown in Figures 4C,D, neurons in the hypoxia group had larger Ca^2+^ currents than neurons in the control group (*P* < 0.05 or < 0.01). Moreover, GB reduced the magnitudes of the Ca^2+^ currents in hypoxic neurons (*P* < 0.05 or 0.01), suggesting that GB may restrict Ca^2+^ influx in neurons exposed to hypoxia.

### GB May Restrain [Ca^2+^]_i_ in Hypoxic Neurons by Inhibiting Ca^2+^ Influx and Intracellular Ca^2+^ Release

The Na^+^-Ca^2+^ exchanger, voltage-dependent Ca^2+^ channels and glutamate receptor channels are the main pathways participating in Ca^2+^ influx in neurons (Blaustein and Lederer, 1999). Furthermore, Ca^2+^ release from the ER and mitochondria may also contribute to Ca^2+^ overload in neuronal cells (Bodalia et al., 2013). Therefore, we evaluated the mRNA and protein expressions of the genes encoding Ca_v_1.2, STIM1 and RyR2, all of which are known to have important roles in neuronal Ca^2+^ regulation and high expression in neurons. L-type Ca^2+^ channels (LTCC) are mainly composed of Cav1.2 subunits in neurons (Liu et al., 2018). STIM1 (Stromal interaction molecule 1) is a highly conserved sensor of calcium concentration in the endoplasmic reticulum (ER) and ubiquitously expressed in different tissues (Williams et al., 2001). RyR2 (type-2 ryanodine receptor) is an ER channel mediating Ca^2+^ release which express predominating in brain and exerts critical roles in hippocampal synaptic plasticity and spatial memory processes (More et al., 2018).

**Figure 5:**
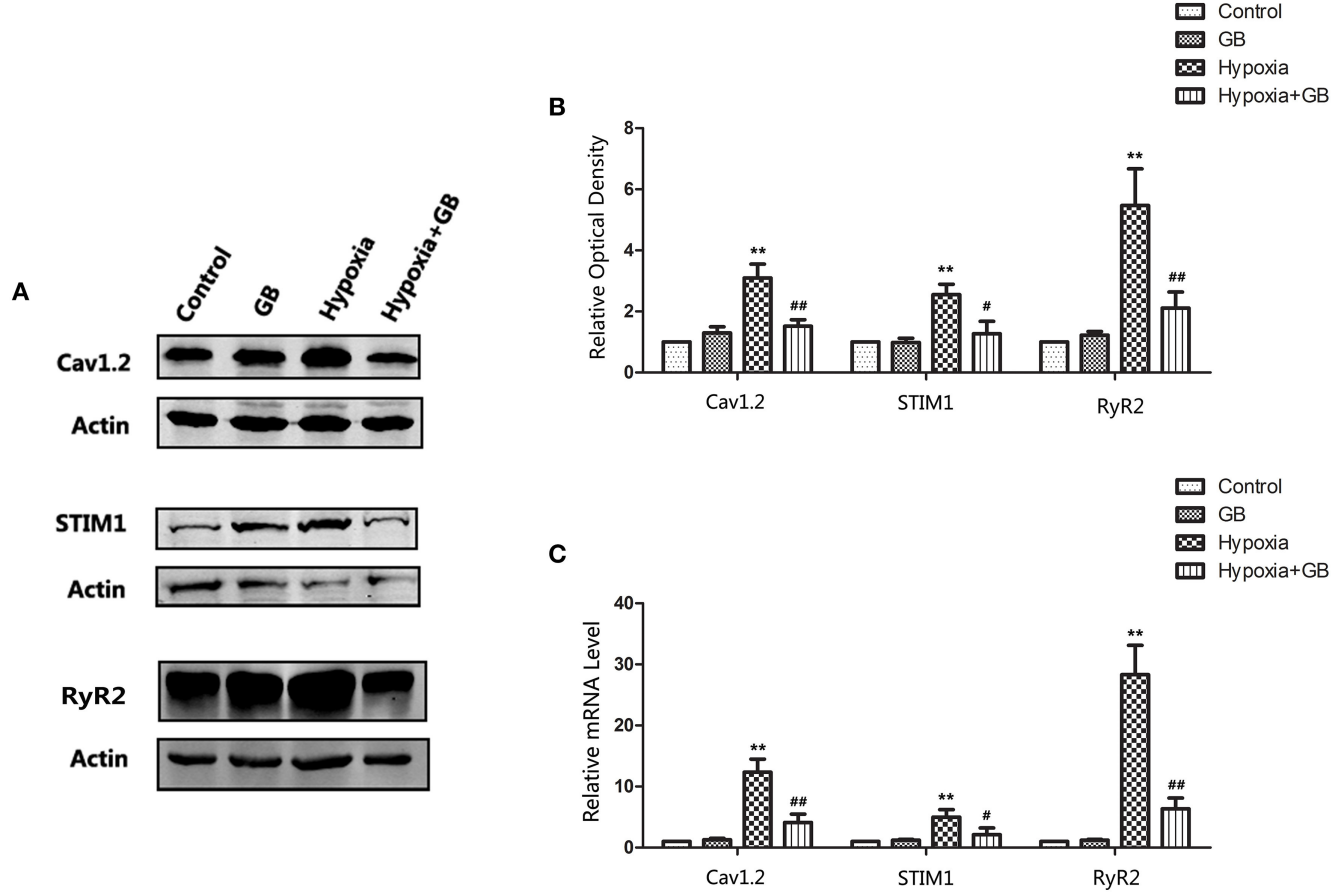
GB reduced the expressions of proteins involved in intracellular calcium homeostasis in neurons exposed to hypoxia. **(A)** Representative western blots showing the expressions of Ca_v_1.2, STIM1 and RyR2 proteins in hippocampal tissue from the various groups. **(B)** Quantification of the western blot data. **(C)** qPCR analysis of the mRNA levels of Ca_v_1.2, STIM1 and RyR2. Datafor **(B)** and **(C)** are expressed as the mean ± SEM (*n* = 3). **P < 0.01 for hypoxia group vs. control group; ^#^*P* < 0.05, ^##^*P* < 0.01 for hypoxia-GB group vs. hypoxia group (Student's *t*-test).

As shown in Figure 5, the protein and mRNA expressions of Ca_v_1.2, STIM1 and RyR2 were increased in neurons of the hypoxia group (*P* < 0.01 vs. control), but these effects of hypoxia were significantly attenuated by GB (*P* < 0.05). The administration of GB alone (in the absence of hypoxia) did not affect the expression of protein and mRNA. Taken together, the above results support the inference that hypoxia enhances Ca ^2+^ influx and intracellular Ca^2+^ release and that GB suppresses these effects to maintain Ca^2+^ homeostasis.

## Discussion

The principal findings of the present study are: (1) GB enhanced the cellular metabolic activity of hypoxic hippocampal neurons; (2) GB lowered [Ca^2+^]_i_ in neurons exposed to hypoxia; (3) GB decreased the frequency of spontaneous discharges and the number of action potentials in hypoxic neurons; (4) GB suppressed the magnitude of the Ca^2+^ current in hypoxic neurons; and (5) under hypoxic conditions, GB reduced the mRNA and protein expressions of the genes encoding Ca_v_1.2, STIM1 and RyR2, which are known to play important roles in neuronal Ca^2+^ homeostasis. Taken together, the above findings suggest that hypoxia may induce Ca^2+^ overload in neuronal cells and that GB may protect against hypoxia-induced neuronal cell damage by maintaining Ca^2+^ homeostasis, possibly through reductions in Ca^2+^ entry through voltage-dependent Ca^2+^ channels and store-operated channels and inhibition of Ca^2+^ release from intracellular stores.

A notable finding of the present study was that GB increased the cellular metabolic activity of cultured hippocampal neurons exposed to chemical hypoxia. Similar protective effects of GB against hypoxia have been described previously in animal models of ischemic stroke. For example, GB was reported to reduce infarct volume and neurological deficits in mice with middle cerebral artery occlusion, and the suggested mechanisms included heme oxygenase-1, upregulation of brain-derived neurotrophic factor expression, modulation of microglia/macrophages, inhibition of nuclear factor kappa-B-induced inflammation and apoptosis, suppression of oxidative damage, and improved blood-brain-barrier function (Lv et al., 2011; Shah et al., 2011; Gu et al., 2012; Huang et al., 2012; Shu et al., 2016; Wei et al., 2017). Thus, there is mounting evidence that GB can attenuate cerebral injury following hypoxia.

Hypoxia *in vivo* and *in vitro* has been extensively studied as a mechanism of injury in neurons injures (Mitroshina et al., 2018; Fang et al., 2019). An important event associated with hypoxia is the accumulation of intracellular Ca^2+^ due to increased Ca^2+^ entry and disruption of other Ca^2+^ homeostatic mechanisms (Yao and Haddad, 2004), and this accumulation of Ca^2+^ can lead to neurodegeneration, necrosis or apoptosis. Previous studies have provided evidence that components of *Ginkgo biloba* extract such as ginkgolide A, GB and EGb 761 can inhibit Ca^2+^ overload during ischemia (Zhang et al., 2003), upregulate the mRNA expression of calbindin D28K (an intracellular Ca^2+^ buffer), and decrease the intracellular Ca^2+^ concentration (Meng et al., 2007). In our study, pre-treatment with GB significantly decreased [Ca^2+^]_i_ in hypoxic neurons, indicating that GB may attenuate the rise in cytoplasmic Ca^2+^ concentration that invariably occurs in response to a hypoxic insult. In addition, whole-cell patch-clamp recordings revealed that spontaneous discharge frequency and action potential frequency were increased in hypoxic neurons, and these effects of hypoxia were suppressed by pre-treatment with GB. These data indicate that GB may attenuate the increase in neuronal excitability induced by hypoxia, which in turn might contribute to a reduction in excitotoxicity. Importantly, we also observed an increase in the magnitude of the Ca^2+^ current in hypoxic neurons that was partially reversed by GB. This latter finding is consistent with a previous report that GB could inhibit the abnormal voltage-dependent Ca^2+^ current induced by β-amyloid peptide in hippocampal CA1 pyramidal neurons isolated from the rat (Chen et al., 2006). These results imply that GB may limit Ca^2+^ influx in hypoxic neurons.

It is known that the Na^+^-Ca^2+^ exchanger, voltage-dependent Ca^2+^ channels and glutamate receptor channels are the main pathways participating in neuronal Ca^2+^ influx (Hoyt et al., 1998; Aarts et al., 2003). Furthermore, Ca^2+^ release from the ER and mitochondria also contributes to Ca^2+^ overload in hypoxic neuronal cells. As many candidate molecules may be involved in the regulation of these processes, we selected three representative molecules for preliminary study: Ca_v_1.2 (an L-type voltage-gated calcium channel subtype) (Catterall, 2011), STIM1 (a sensor of ER Ca^2+^ that serves to activate store-operated Ca^2+^ entry) (Roos et al., 2005), and RyR2 (responsible for regulation of Ca^2+^ release from the sarcoplasmic reticulum into the cytosol) (Meissner, 1994). Western blot analysis showed that hypoxia was associated with significant increases in the mRNA and protein expressions of Ca_v_1.2, STIM1 and RyR2, and this upregulated expression in hypoxic neurons was inhibited by pre-treatment with GB. These novel findings suggest that GB may regulate calcium homeostasis at several levels.

This study has some limitations. Although we identified changes in the expression of Ca_v_1.2, STIM1 and RyR2 in hypoxic neurons and a corresponding suppression of these changes in neurons pretreated with GB, we did not identify the specific mechanism(s) underlying these effects of GB. Furthermore, it was beyond the scope of this study to elucidate the detailed pathways through which GB influenced Ca^2+^ homeostasis. Additionally, we used 0.4 mM GB *in vitro* and 12 mg/kg GB *in vivo*, but we did not fully evaluate whether there were dose-dependent effects of GB on changes in intracellular Ca^2+^ concentration. Nevertheless, our results form an important basis for future research to address these issues.

## Conclusion

Taken together, the findings of our study suggest that GB protects hippocampal neurons from hypoxia at least in part via the regulation of calcium homeostasis. Future studies will address the specific mechanisms and signaling cascades underlying this protective effect.

## Data Availability Statement

The raw data supporting the conclusions of this article will be made available by the authors, without undue reservation.

## Ethics Statement

The animal study was reviewed and approved by The Ethics Committee for Animal Experiments of the Fourth Military Medical University.

## Author Contributions

JF and QS were corresponding author and they were responsible for experimental design. LW and QL performed sample preparation, establishment of an *in vivo* and *in vitro* model of hypoxia, confocal fluorescence microscopy and electrophysiology experiments. SZ and WX performed all calculation and data analysis. WD and JR were responsible for real-time PCR and WB. All authors read and contributed to the manuscript.

## Conflict of Interest

The authors declare that the research was conducted in the absence of any commercial or financial relationships that could be construed as a potential conflict of interest.
